# Pseudomonas beijingensis sp. nov., a novel species widely colonizing plant rhizosphere

**DOI:** 10.1099/ijsem.0.006473

**Published:** 2024-07-26

**Authors:** Kaiji Liao, Jingyi Wu, Can Wang, Jun-Zhou Li, Hai-Lei Wei

**Affiliations:** 1 State Key Laboratory of Efficient Utilization of Arid and Semi-arid Arable Land in Northern China, Key Laboratory of Microbial Resources Collection and Preservation, Ministry of Agriculture and Rural Affairs, Institute of Agricultural Resources and Regional Planning, Chinese Academy of Agricultural Sciences, Beijing 100081, PR China

**Keywords:** novel species, plant growth-promoting rhizobacteria, polyphasic taxonomy, *Pseudomonas*

## Abstract

A polyphasic taxonomic approach was used to characterize the three bacterial strains (FP830^T^, FP2034, and FP2262) isolated from the rhizosphere soil of rice, corn, and highland barley in Beijing, Heilongjiang, and Tibet, respectively, in PR China. These strains were Gram-negative, rod-shaped, and have one or two polar flagella. They exhibited optimal growth at 28 °C and pH 7.0 in the presence of 1 % (w/v) NaCl and showed fluorescence under ultraviolet light when cultivated on King’s B plates. The FP830^T^ genome size is 6.4 Mbp with a G+C content of 61.0 mol%. FP830^T^ has the potential to promote plant growth by producing various metabolites such as fengycin, pyoverdin, indole-3-acetic acid, and the volatile substance 2,3-butanediol. Phylogenetic analysis indicated that three isolates formed an independent branch, which most closely related to type strains *Pseudomonas thivervalensis* DSM 13194^T^ and *Pseudomonas zanjanensis* SWRI12^T^. The values of average nucleotide identity and digital DNA–DNA hybridization between three isolates and closest relatives were not higher than 93.7 and 52.3 %, respectively. The dominant cellular fatty acids were C_16 : 0_, summed feature 3 (C_16 : 1_* ω*7*c*/C_16 : 1_ ω6*c*), and summed feature 8 (C_18 : 1 _ω7*c*/C_18 : 1 _ω6*c*). The major polar lipids were phosphatidylethanolamine, diphosphatidylglycerol, and aminophospholipid. The predominant respiratory quinone was ubiquinone (Q-9). Based on polyphasic taxonomic analysis, it was concluded that strains FP830^T^, FP2034, and FP2262 represented a novel species within the genus *Pseudomonas*, and *Pseudomonas beijingensis* sp. nov. was proposed for the name of novel species. The type strain is FP830^T^ (=ACCC 62448^T^=JCM 35689^T^).

## Introduction

The genus *Pseudomonas* comprises many species and belongs to the family *Pseudomonadaceae* within the class *Gammaproteobacteria* [1]. These bacteria are Gram-negative, non-spore-forming, aerobic, motile, and rod-shaped with one or several polar flagella [2,3]. They exhibit great metabolic diversity and can colonize a wide range of ecological niches, such as soil, marine environments, plants, animals, tar pits, and glaciers [4,5]. *Pseudomonas* isolates are commonly employed as plant growth-promoting rhizobacteria due to their ability to produce 2,4-diacetylphlorglucinol [6], polyphenolic compounds [7], lipopeptides [8], indole-3-acetic acid (IAA) [9] and 2,3-butanediol [10], as well as inducing an immune response [11].

Based on the List of Prokaryotic names with Standing in Nomenclature (https://lpsn.dsmz.de/), the genus *Pseudomonas* consists of around 330 species with a validly published and correct name [12]. *Pseudomonas* species have been categoried into 11–13 groups, with the most extensive group being *P. fluorescens*, which can be further subdivided 8–9 subgroups [13,14]. For bacterial classification, the Genome Taxonomy Database offers a prokaryotic taxonomy based on genome sequences. In this database, two large taxonomic categories, *Pseudomonas* and *Pseudomonas* E, provide comprehensive genome information, including most of isolated and uncultured *Pseudomonas* [15].

Here, we focused on strains FP830^T^, FP2034, and FP2262 from the rhizospheres of three distinct plants from various areas. These isolates were identified and classified using a polyphasic taxonomic approach that included phenotypic, chemotaxonomic, phylogenetic, and genomic analysis [16]. The results revealed that these strains represent a novel species within the genus *Pseudomonas*.

## Isolation and ecology

Strains FP830^T^, FP2034, and FP2262 were isolated from the rhizosphere of rice, corn, and highland barley plants collected from different locations in PR China: Haidian District, Beijing (40° 13′ 51.12″ N 116° 18′ 37.97″ E); Qiqihaer City, Heilongjiang Province (48° 55′ 87.97″ N 125° 55′ 94.97″ E); and Linzhi City, Tibet (29°47′ 54.64″ N 94° 41′ 70.34″ E), respectively. To isolate bacteria, 1 g soil was suspended in 9 ml sterile water and incubated at 28 °C with shaking at 200 r.p.m. for 1 h. Serial dilutions of the suspensions of sterile water were spread on King’s B (KB) agar medium (20 g l^−1^ Bacto tryptone, 1.5 g l^−1^ MgCl_2_, 1.5 g l^−1^ K_2_HPO_4_, and 1.5 % (v/v) glycerol), and aerobically incubated at 28 °C for 24 h. Colonies displaying fluorescence under UV illumination at 254 nm were selected and purified three times using the same media. Strains FP830^T^, FP2034, and FP2262 were stored at −80 °C in a 20 % (v/v) aqueous glycerol solution for long-term preservation.

## Molecular identification

To verify the taxonomic status of the strains, we amplified the 16S rRNA gene sequences of three strains with the primers 27F and 1492R [17] and sequenced them using Sanger sequencing. The nearly complete 16S rRNA gene sequences (1386 bp) were first compared to the EzBioCloud database to identify closely related species [18], which were downloaded from EzBioCloud’s Identify Service and used to reconstruct the phylogenctic tree. Sequence alignment was performed using muscle and a maximum-likelihood phylogenetic tree was reconstructed based on the Kimura two-parameter model with mega X [19,20]. Confidence in tree topology was determined using 1000 bootstrap resamplings. Intergenomic distances were calculated with the Genome blast Distance Phylogeny method under the algorithm 'coverage' and distance formula *d5* [21]. The phylogenomic tree was reconstructed using FastME followed by subtree pruning and regrafting post-processing [22]. Branch support was inferred from 1000 pseudobootstrap repetitions. Genome sequences of closely related type strains were obtained from the GenBank. Genome-to-genome computational comparisons were performed between pairwise strains. The average nucleotide identity (ANI) values were obtained using the EzBioCloud web service (https://www.ezbiocloud.net/tools/ani) [23]. The digital DNA–DNA hybridization (dDDH) values were determined using the Genome-to-Genome Distance Calculator [24].

**Fig. 1.**
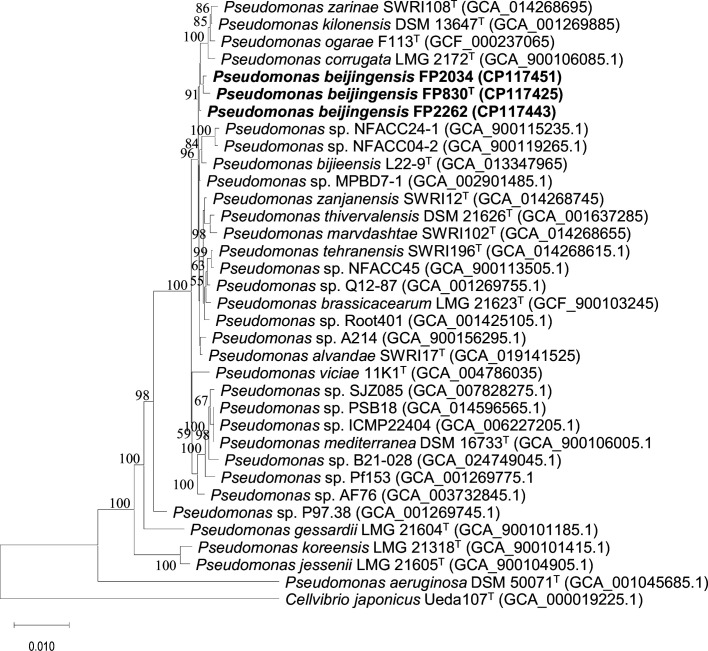
Phylogenetic tree based on the 16S rRNA gene sequences, demonstrating the relationships between strains FP830^T^, FP2034, and FP2262 and related species of the genus *Pseudomonas*. Dendrograms were generated by the maximum-likelihood method. Bootstrap values >50 % (based on 1000 resamplings) are shown, Bar, 1 nt substitution per 100 nt. The superscript T denotes the type strain of the species used, and the GenBank accession number of each strain is in parentheses.

**Fig. 2.**
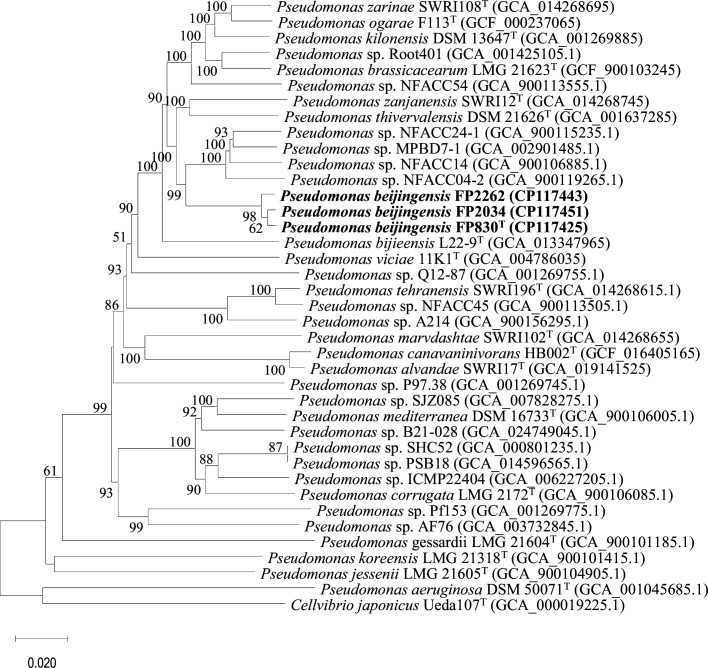
Phylogenetic tree based on the whole genome sequence of three isolates and closely related species of the genus *Pseudomonas*. Pairwise comparisons among genomes were conducted using genome blast distance phylogeny. The resulting intergenomic distances were used to infer a balanced minimum-evolution tree via FastME. Pseudo-bootstrap values>50 % based on 1000 replications are indicated on branches. Bar, 2 nt substitution per 100 nt. The superscript T denotes the type strain of the species used, and the GenBank accession number of each strain is in parentheses.

Comparing the 16S rRNA gene sequences of FP830^T^, FP2262, and FP2034 with the EzBioCloud database revealed that *P. thivervalensis* DSM 13194^T^ was the most closely related species, with a 99.8 % sequence similarity (Table S1, available in the online Supplementary Material). The phylogenetic analysis of the 16S rRNA sequences showed that the three isolates belonged to the genus *Pseudomonas*, and had a recent common ancestor with *P. corrugata* LMG 2172^T^, *P. ogarae* F113^T^, *P. kilonensis* DSM 13647^T^, and *P. zarinae* SWRI108^T^ (Fig. 1). To improve the taxonomic resolution between closely related interspecies, we performed phylogenomic analyses based on whole genomes. The phylogenomic tree showed that FP830^T^, FP2034, and FP2262 clustered into a separate branch and formed a sister group with unnamed strains *Pseudomonas* sp. NFACC04-2, NFACC14, MPBD7-1, and NFACC24-1, while having a recent common ancestor with the type strains *P. thivervalensis* DSM 13194^T^ and *Pseudomonas zanjanensis* SWRI12^T^ (Fig. 2). All trees indicated that FP830^T^, FP2034, and FP2262 belonged to the *P. corrugata* subgroup. Among the closely related species, pairwise ANI values ranged from 87.1 (*P*. sp. Pf153) to 93.6 % (*P*. sp. MPBD7-1), with the closest value to the type strain being 93.1 % (*P. thivervalensis* DSM 13194^T^). All ANI values fell below the widely accepted species delineation threshold 95–96 % [23]. In addition, the pairwise dDDH values ranged from 32.0 (*P*. sp. Pf153) to 52.3 % (*P*. sp. MPBD7-1), with the closest value to the type strain being 49.8 % (*P. thivervalensis* DSM 13194^T^), both of which were less than the 70 % cut–off for species determination [24] (Table 1). These data indicated that the three isolates represent a new species of *Pseudomonas*.

**Table 1.** Whole genome relatedness analysis of FP830^T^, FP2034, and FP2262 and related species from the genus *Pseudomonas* The average nucleotide identity (ANI) and digital DNA–DNA hybridization (dDDH) values were calculated with online services at https://www.ezbiocloud.net/tools/ani and http://ggdc.dsmz.de/distcalc2.php, respectively. The data shown here are sorted in descending order of their dDDH scores against the FP830^T^.

Species	ANI (%)			DDH (%)		
	FP830^T^	FP2034	FP2262	FP830^T^	FP2034	FP2262
*P. beijingensis* FP830^T^	100	99.5	99.1	100	95.4	91.0
*P. beijingensis* FP2034	99.5	100	99.1	95.4	100	91.6
*P. beijingensis* FP2262	99.1	99.1	100	91.0	91.6	100
*P*. sp. MPBD7-1	93.6	93.6	93.7	52.0	52.1	52.3
*P*. sp. NFACC24-1	93.4	93.4	93.4	50.9	50.8	51.1
*P*. sp. NFACC14	93.3	93.3	93.3	50.6	50.4	50.9
*P*. sp. NFACC04-2	93.3	93.2	93.3	49.9	49.9	50.2
*P. thivervalensis* DSM 21626^T^	93.0	93.1	93.1	49.7	49.8	49.8
*P. zarinae* SWRI108^T^	92.6	92.7	92.6	47.7	47.7	47.7
*P. ogarae* F113^T^	92.6	92.6	92.6	47.6	47.7	47.7
*P. kilonensis* DSM 13647^T^	92.6	92.7	92.6	47.6	47.6	47.6
*P. zanjanensis* SWRI12^T^	92.8	92.7	92.8	47.4	47.4	47.6
*P*. sp. Root401	92.5	92.6	92.5	47.3	47.4	47.2
*P. brassicacearum* LMG 21623^T^	92.4	92.5	92.4	47.0	47.0	47.2
*P. bijieensis* L22-9^T^	92.0	92.0	92.0	45.0	45.1	45.1
*P*. sp. NFACC54	91.8	91.9	91.9	44.5	44.6	44.7
*P. viciae* 11K1^T^	90.0	90.1	90.0	39.2	39.3	39.2
*P*. sp. Q12-87	88.9	88.9	88.9	35.7	35.7	35.9
*P. marvdashtae* SWRI102^T^	88.3	88.4	88.4	35.6	35.6	35.6
*P*. sp. A214	88.7	88.6	88.7	35.2	35.4	35.3
*P. tehranensis* SWRI196^T^	88.1	88.2	88.0	34.7	34.8	34.7
*P. alvandae* SWRI17^T^	88.5	88.5	88.6	34.6	34.6	34.6
*P*. sp. NFACC45	88.3	88.2	88.2	34.5	34.5	34.5
*P. canavaninivorans* HB002^T^	88.5	88.5	88.6	34.4	34.5	34.4
*P*. sp. P97.38	88.5	88.4	88.4	34.2	34.3	34.3
*P*. sp. B21-028	87.8	87.8	87.8	33.3	33.4	33.4
*P*. sp. SHC52	87.7	87.7	87.7	33.3	33.4	33.3
*P*. sp. PSB18	87.8	87.8	87.7	33.3	33.3	33.2
*P. mediterranea* DSM 16733^T^	87.9	87.8	87.8	33.2	33.2	33.2
*P*. sp. ICMP22404	87.7	87.8	87.7	33.1	33.2	33.1
*P*. sp. SJZ085	87.6	87.6	87.5	33.1	33.1	33.1
*P*. sp. AF76	87.7	87.6	87.6	33.1	33.1	33.0
*P. corrugata* LMG 2172^T^	87.4	87.4	87.3	32.6	32.6	32.6
*P*. sp. Pf153	87.1	87.1	87.1	32.1	32.1	32.0
*P. koreensis* LMG 21318^T^	84.5	84.4	84.4	26.9	26.9	26.9
*P. jessenii* LMG 21605^T^	84.8	84.8	84.8	26.9	26.8	26.9
*P. gessardii* LMG 21604^T^	83.8	83.7	83.8	25.8	25.8	25.8
*P. aeruginosa* DSM 50071^T^	79.9	80.0	79.8	21.1	21.2	21.2

## Genome features and mining

Bacterial DNA was extracted from each culture using the Bacterial Genomic DNA Extraction Kit (D1600, Solarbio) according to the manufacturer’s instructions. Genome sequencing was performed using the Illumina HiSeq and PacBio RSII platforms. After quality determination using NanoDrop2000, genomic DNA libraries were prepared for Illumina and PacBio sequencing. The two platforms generated approximately 2.0 Gb data. HGAP4 was used to assemble the *de novo* PacBio long reads [25]. The genome was polished using Pilon for three rounds [26]. Genome assemblies were evaluated using quast [27]. The protein coding sequences (CDS) of each genome were predicted using Prodigal [28], and annotated using the Basic Local Alignment Search Tool (blast) by searching the NCBI non-redundant (NR) database [29]. The rRNA and tRNA were predicted by RNAmmer and tRNAScan-SE [30,31], respectively. Functional annotation was performed using Cluster of Orthologous Groups of proteins (COG) database [32]. The carbohydrate active enzymes (CAZy) genes were identified using blastp against the CAZy database [33]. The analysis of biosynthetic gene clusters (BGCs) for secondary metabolites was performed using antiSMASH with default settings [34]. Protein secretion systems were identified using MacSyFinder [35]. Other ecologically relevant genes were obtained by blast against known sequences from UniProt.

The genomic information of the three isolates is summarized in Table S2. In total, 5384 predicted genes were annotated to 23 categories in FP830^T^. Among these categories, the ‘general function prediction only’ (COG R) category represents the largest group (647, 12.0 %), followed by ‘amino acid transport and metabolism’ (COG E; 584, 10.8 %) and ‘transcription’ (COG K; 480, 8.9 %). The categories ‘RNA processing and modification’ (COG A; 1, 0 %), ‘chromatin structure and dynamics’ (COG B; 2, 0 %), and ‘extracellular structures’ (COG W; 3, 0 %) were the smallest groups (Fig. S1). FP830^T^ contained 527 gene counts distributed unequally among glycosyl transferases (GTs, 44.2 %), glycoside hydrolases (GHs, 30.4 %), carbohydrate binding modules (CMBs, 11.2 %), carbohydrate esterases (CEs, 9.7 %), auxiliary activities (AAs, 3.2 %), and polysaccharide lyases (PLs, 1.3 %) (Fig. S2). AntiSMASH identified 13 BGCs, nine of which were similar to known BGCs. Some of them such as fragin, fengycin and lankacidin C have been reported to have antagonistic activities against plant pathogens, and some of them such as pyoverdin can competitively uptake iron from the environment [36,39]. However, more research is needed to determine whether *P. beijingensis* produces such antimicrobial compounds (Table S3). In addition, we discovered genes encoding tryptophan-2-monooxygenase (IaaM) and indole3-acetamide (IAM) hydrolase (IaaH), two enzymes involving in IAA synthesis. We also found some genes involved in the pyrroloquinoline quinone (PQQ), a redox cofactor involved in phosphate solubilization, as well as genes involved in the formation of volatile chemicals such as 2,3-butanediol and acetoin (Table S4). Interestingly, the FP830^T^ genome has various protein secretion systems, including the type III secretion system (T3SS) (Table S4). T3SS is a primary virulence factor in pathogenic bacteria, such as *P. syringae* [40]. However, T3SS was also found in some non-pathogenic bacteria. Although the role of T3SS in plant rhizosphere *P. fluorescens* remains largely unknown, it has been revealed that T3SS in some *P. fluorescens*, such as *P. fluorescens* C7R12 [41]and *P. fluorescens* KD [42] contributes to beneficial effects on plants. It is a promising topic to investigate the roles of T3SS in the interactions between strain FP830 and plants.

## Physiology and chemotaxonomy

**Fig. 3.**
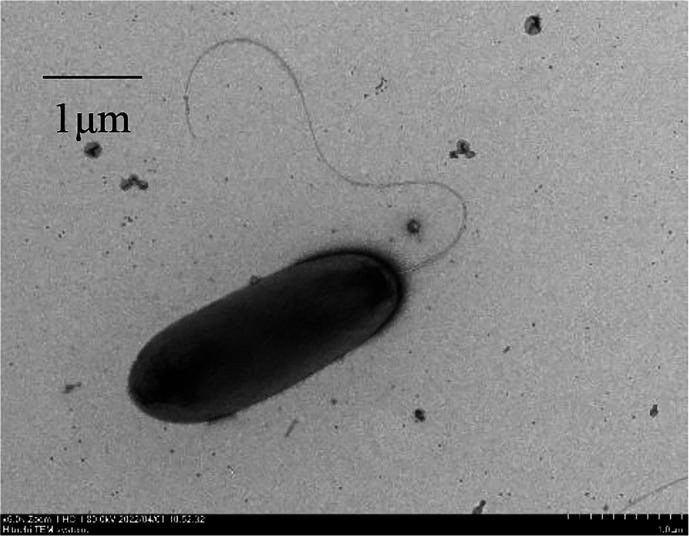
Cell morphology of strain FP830^T^ captured by transmission electron microscopy. Scale bar, 1 µm.

Cell morphology and size were observed using a transmission electron microscope (Hitachi HT7700) after overnight incubation in tryptic soy agar (TSA) medium at 28 ℃. Colony appearance was examined on TSA medium after incubation for 24 h under optimal growth conditions. Growth was tested on TSA at different temperatures (2, 4, 15, 25, 28, 30, 32, 37, 39, 41, and 43 ℃), pH (pH 4–11, at 1 pH unit intervals), and NaCl concentrations (0–11 %, w/v, at 1 % intervals). The buffer systems for adjusting pH was prepared according to a previous report (pH 4.0–5.0, 0.1 M sodium acetate–0.1 M acetic acid; pH 6.0–8.0, 0.1 M KH_2_PO_4_–0.1 M NaOH; pH 9.0–10.0, 0.1 M NaHCO_3_–0.1 M Na_2_CO_3_; pH 11.0, 0.05 M Na_2_HPO_4_–0.1 M NaOH) [43]. Production of fluorescent pigments was detected under UV illumination at 254 nm on KB agar plate [44]. Gram staining was performed using a Gram-staining kit (Solarbio) according to the manufacturer’s instructions. Cells of FP830^T^ were Gram-negative, rod-shaped (0.8–1.0×2.5–3.0 µm) with one or two polar flagella (Fig. 3). Colonies were circular with entire margins, pale yellow, smooth, and slightly convex. The culture produced fluorescent pigments on KB medium. The temperature range for growth was 4–37 °C with an optimum temperature at 28 °C, the pH range was from pH 6 to 9 with an optimum at pH 7, and the NaCl concentration range was 0–4 % (w/v) with an optimum at 1 % (w/v).

Biochemical and physiological characteristics were examined using an API 20NE system (bioMérieux). Carbon source utilization and sensitivity to various chemicals were assessed using the Biolog GEN III MicroPlate system. All physiological and biochemical analyses were perfomed after 4 days of incubation at 28 °C on TSA medium. Table 2 summarizes the key physiological and biochemical characteristics that distinguish strain FP830^T^ from closely related species that were tested in this study, except for the traits of *P. viciae* KACC 21650^T^ that were cited from the literature [45]. Notably, unlike than other species, FP830^T^ could utilize d-galacturonic acid.

**Table 2.** Characteristics that differentiate strain *P. beijingensis* FP830^T^ from closely related *Pseudomonas* type strains Strain: 1, FP830^T^; 2, *P*. *thivervalensis* DSM 13194^T^; 3, *P*. *kilonensis* DSM 13647^T^; 4, *P*. *brassicacearum* DSM 13227^T^; 5, *P*. *viciae* KACC 21650^T^. +, Positive; −, negative; w, weak; nd, no data available.

Characteristics	1	2	3	4	5
**API 20 NE tests**					
l-Arginine	−	w	−	−	−
Gelatin	−	+	−	+	−
*N*-Acetyl glucosamine	+	+	+	+	−
Gluconate	+	+	+	+	w
**Carbon source utilization assays (Biolog GENIII)**					
Sucrose	−	−	+	w	+
d-Fructose	+	+	+	w	w
d-Fucose	+	+	−	w	w
d-Aspartic acid	+	−	−	+	+
d-Serine	−	+	−	+	w
Minocycline	+	−	−	−	w
l-Glutamic acid	w	−	+	+	nd
Pectin	−	−	+	−	+
d-Galacturonic acid	+	−	−	−	w
l-Galactonic acid lactone	+	+	−	+	w
d-Gluconic acid	−	−	+	+	+
d-Glucuronic acid	+	+	−	−	w
Glucuronamide	+	+	−	w	w
Mucic acid	+	w	+	−	+
l-Lactic acid	−	−	+	−	+
α-Keto-glutaric acid	−	−	+	−	+
γ-Amino-butryric acid	+	−	+	w	+
Aztreonam	+	+	+	w	w

To analyse cellular fatty acids, polar lipids, and respiratory quinones, cells of the FP830^T^ and reference strains were harvested from TSA broth after incubation for 1 day at 28 °C. Cellular fatty acids were analysed after converting them into fatty acid methyl esters by saponification, methylation, and extraction using the midi Microbial Identification System Sherlock version 6.2 and the TSBA6 database [46,47]. Major polar lipids were were analysed by two-dimensional TLC [48]. Isoprenoid quinones were extracted with a chloroform–methanol (2 : 1, v/v) mixture, and analysed by HPLC as described previously [49]. The major fatty acids in strain FP830^T^ were summed feature 3 (C_16 : 1_* ω*7*c*/C_16 : 1_ ω6*c*), C_16 : 0_, and summed feature 8 (C_18 : 1_* ω*7*c*/C_18 : 1_ ω6*c*) making up more than 66.8 % of the total (Table 3). The major polar lipids in FP830^T^ were phosphatidylethanolamine, diphosphatidylglycerol, and aminophospholipid (Fig. S3). The results of isoprenoid quinone analysis indicated that the predominant respiratory quinone of strain FP830^T^ was ubiquinone 9 (Fig. S4).

**Table 3.** Cellular fatty acid composition of strain FP830^T^ and the type strains of closely related *Pseudomonas* species Strain: 1, FP830^T^; 2, *P. thivervalensis* DSM 13194^T^; 3, *P. kilonensis* DSM 13647^T^; 4, *P. brassicacearum* DSM 13227^T^; 5, *P. viciae* KACC 21650^T^.

Fatty acid	1	2	3	4	5
Summed features*					
3	30.1	19.6	34.2	23.6	22.4
8	15.2	11.1	15.0	12.3	19.5
**Saturated**					
C_12 : 0_	10.7	4.1	9.7	4.3	3.3
C_16 : 0_	21.5	27.7	25.5	25.8	23.4
**Hydroxy**					
C_10 : 0_ 3-OH	4.6	4.0	4.1	4.0	3.8
C_12 : 0_ 2-OH	2.8	4.7	1.7	5.2	4.7
C_12 : 0_ 3-OH	5.3	5.0	4.4	5.0	4.7
**Cyclo**					
C_17 : 0_ cyclo	7.8	19.4	3.7	17.0	11.8

*Summed Ffeatures are fatty acids that cannot be resolved reliably from another fatty acid using the chromatographic conditions chosen. The MIDImidi system groups these fatty acids together as one feature with a single percentage of the total. Summed feature 3 comprises C_16 : 1_* ω*7*c*/C_16 : 1_ ω6*c* and summed feature 8 comprises C_18 : 1_* ω*7*c*/C_18 : 1_ ω6*c*.

## Description of *Pseudomonas beijingensis* sp. nov.

*Pseudomonas beijingensis* (bei.jing.en’sis. N.L. fem. adj. *beijingensis,* pertaining to Beijing, PR China, where the type strain was isolated).

Cells were Gram-reaction-negative, rod-shaped with one to two polar flagella, 2.5–3 µm long (mean 2.7 µm), and 0.8–1.0 µm wide (mean 0.9 µm). Colonies were round with entire margin, pale yellow, smooth, and slightly convex, after incubation at 28 °C for 2 days on TSA agar plate. Fluorescent pigments were produced when cells were grown on KB agar plates. Growth of the isolate was observed at 4–37 °C (optimum, 28 °C), in the presence of 0–4 % (w/v) NaCl (optimum, 1 %), and at pH 6–9 (optimum, pH 7).

In API 20NE tests, positive responses are observed for aesculin hydrolysis, nitrate reduction, and assimilation of l-arabinose, d-glucose, d-mannose, potassium gluconate, *N*-acetyl-d-glucosamine, malic acid, decanoic acid, and citric acid. In the Biolog GEN III system, positive for the utilization of α-d-glucose, *p*-hydroxy-phenylacetic acid, d-mannose, d-galacturonic acid, γ-amino-butryric acid, d-fructose, l-galactonic acid, lactone, d-galactose, d-glucuronic acid, citric acid, d-fucose, glucuronamide, mucic acid, d-aspartic acid, quinic acid, l-malic acid, acetic acid, and d-serine. Cells exhibited chemical sensitivity to sodium lactate, troleandomycin, lincomycin, vancomycin, nalidixic acid, aztreonam, fusidic acid, tetrazolium violet, lithium chloride, minocycline, niaproof 4, tetrazolium blue, and potassium tellurite. The major fatty acids are C_16 : 0_, summed feature 3 (C_16 : 1_* ω*7*c*/C_16 : 1_ ω6*c*), and summed feature 8 (C_18 : 1_* ω*7*c*/C_18 : 1_ ω6*c)*. The predominant respiratory quinone is ubiquinone 9 (Q-9), and the major polar lipids are phosphatidylethanolamine, diphosphatidylglycerol, and aminophospholipid.

The type strain, FP830^T^ (=ACCC 62448^T^=JCM 35689^T^), was isolated from the rice rhizosphere in Haidian District, Beijing, PR China. The genomic DNA G+C content is 61.0 mol%. GenBank accession numbers for its 16S rRNA and whole genome sequence are OR215004 and CP117425, respectively.

## supplementary material

10.1099/ijsem.0.006473 Uncited Supplementary Material 1.

## Abbreviations

ANI: average nucleotide identity
BGC: biosynthetic gene cluster
CAZy: carbohydrate active enzyme
COG: cluster of orthologous groups
dDDH: digital DNA–DNA hybridization
KB: King’s B
MLSA: multilocus sequence analysis
TSA: tryptic soy agar
