# Supplementary material for: Pseudomonas beijingensis sp. nov., a novel species widely colonizing plant rhizosphere
Source: Int J Syst Evol Microbiol. 2024 Jul 26;74(7):006473. doi: 10.1099/ijsem.0.006473 (PMC11281800; doi:10.1099/ijsem.0.006473)
Supplement: Uncited Supplementary Material 1. [file ijsem-74-06473-s001.pdf]

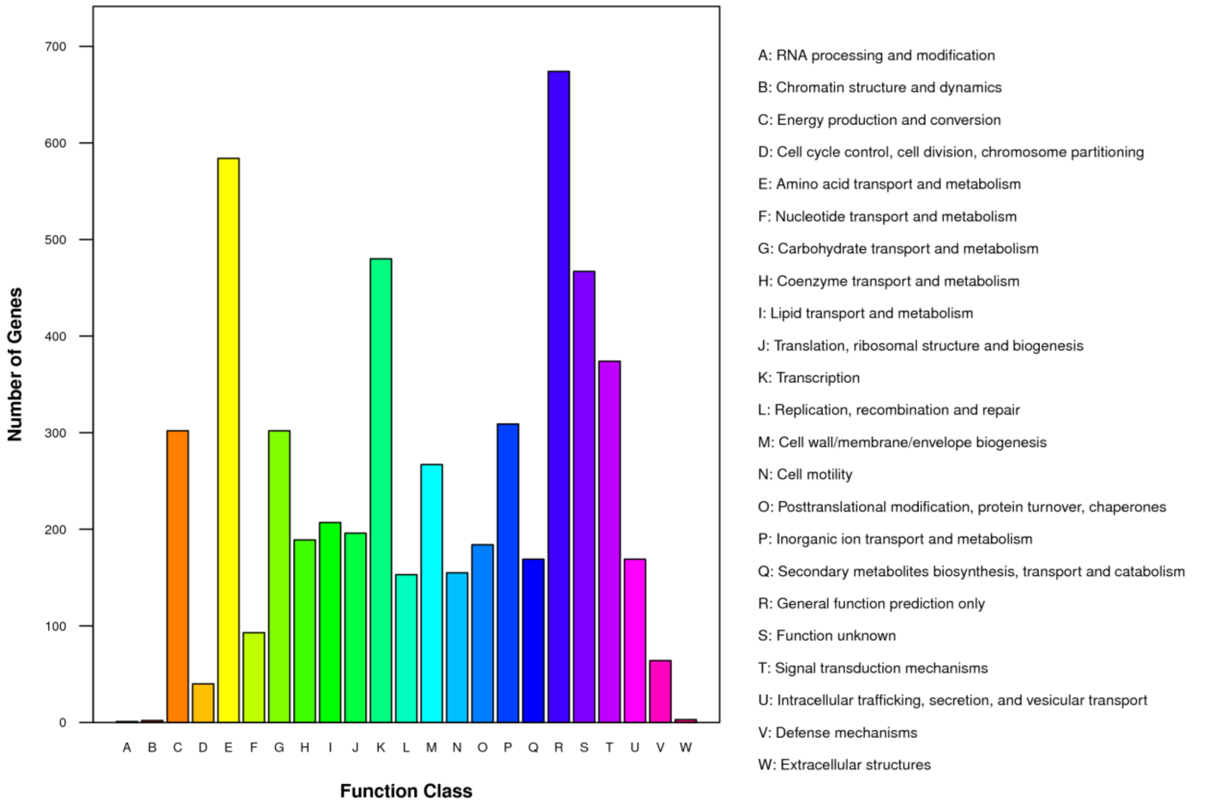

Fig. S1 COG function classification of FP830^{T}.

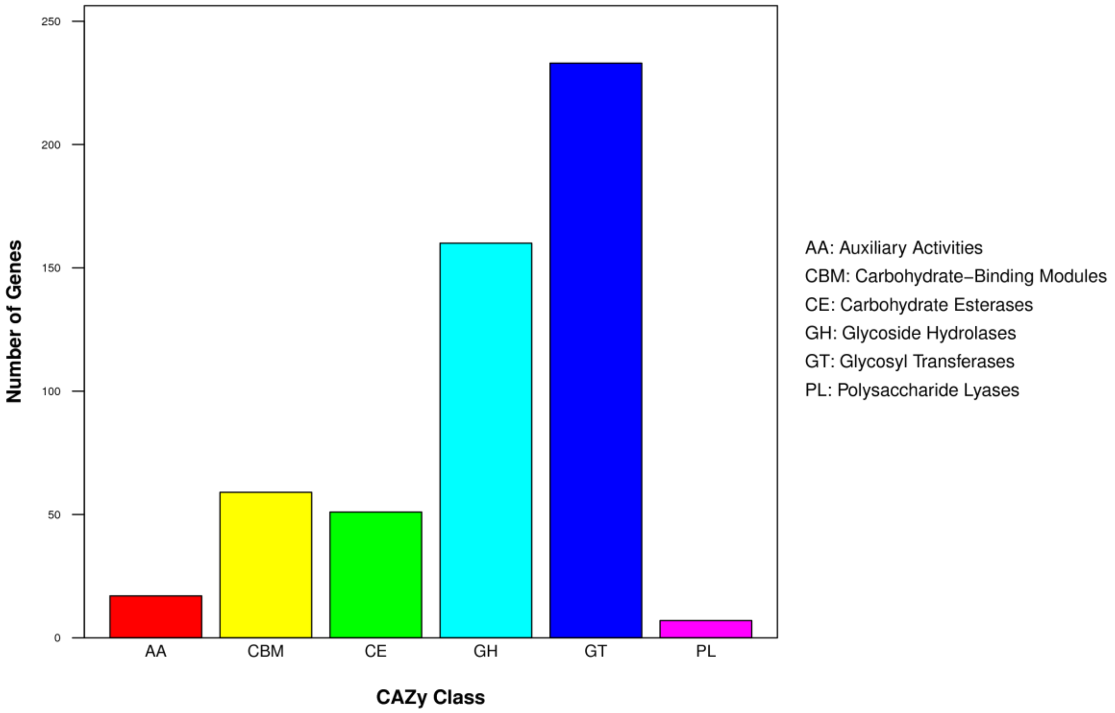

Fig. S2 Carbohydrate-hydrolyzing enzymes in FP830^{T}. Different colors indicate different classes.

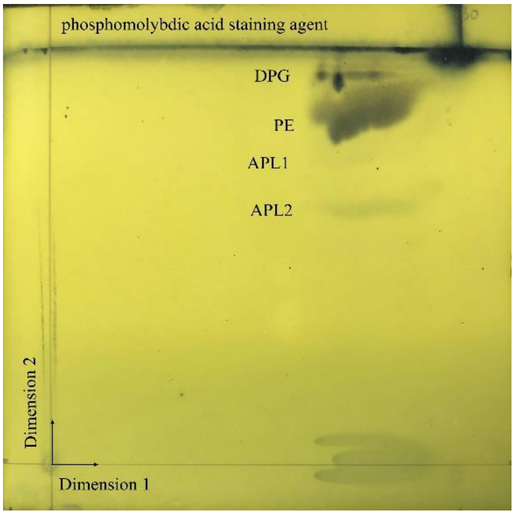

Fig. S3 Two-dimensional TLC plate of polar lipids extracted from *P. beijingensis* FP830^{T}. The plate was sprayed with 10% (v/v) molybdophosphoric acid to detect all the polar lipids present. PE, phosphatidylethanolamine; DPG, diphosphatidylglycerol; APL, aminophospholipid.

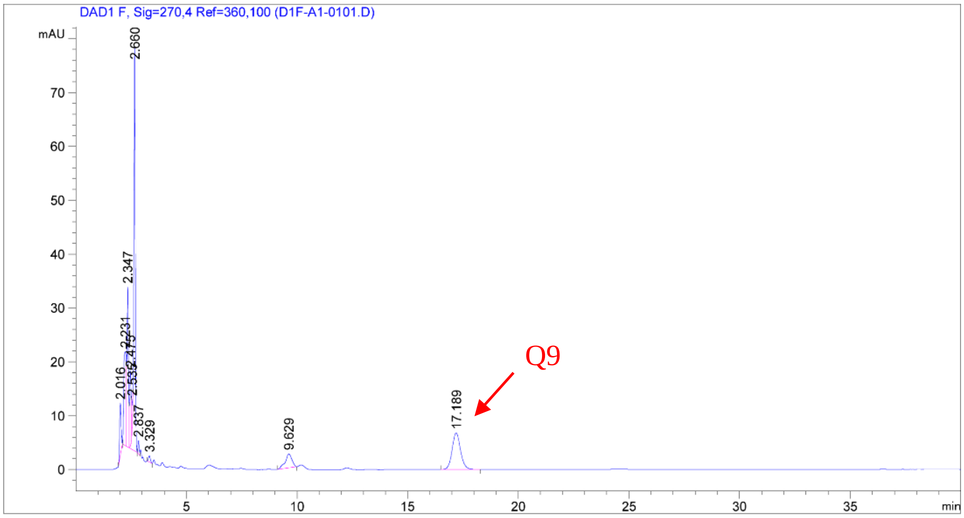

Fig. S4 Respiratory quinone for FP830T. The red arrow indicates the retention time of the respiratory ubiquinone 9.

Table S1. The 16S rRNA sequence similarity between strains FP830^{T}, FP2034, and FP2262 and the type strains of closely related *Pseudomonas* species.

Species	16S rRNA sequence similarity (%)		
	FP830^{T}	FP2034	FP2262
Pseudomonas thivervalensis DSM 13194^{T}	99.8	99.8	99.8
Pseudomonas kilonensis DSM 13647^{T}	99.8	99.8	99.7
Pseudomonas piscium CECT 30175^{T}	99.8	99.8	99.7
Pseudomonas brassicacearum DSM 13227^{T}	99.4	99.7	99.7
Pseudomonas corrugata DSM 7228^{T}	99.6	99.6	99.5
Pseudomonas viciae KACC 21650^{T}	99.5	99.5	99.4
Pseudomonas mediterranea DSM 16733^{T}	99.3	99.3	99.4
Pseudomonas lini DSM 16768^{T}	99.2	99.2	99.2
Pseudomonas kielensis DSM 111668^{T}	99.1	99.1	99.0
Pseudomonas frederiksbergensis DSM 13022^{T}	99.0	99.0	99.0
Pseudomonas arsenicoxydans CECT 7543^{T}	98.9	98.9	98.8
Pseudomonas veronii DSM 11331^{T}	98.8	98.8	98.8
Pseudomonas mandelii ATCC 700871^{T}	98.7	98.7	98.6

Homology searches for related *Pseudomonas* species based on 16S rRNA gene sequences were performed using the EzBioCloud database.

Table S2. Genomic characteristics of strains *P. beijingensis* FP830^T, FP2034 and FP2262.

Strains	Chromosome size (megabase pairs)	Bases (CDSs)	G+C (%)	Protein-coding sequences (CDSs)	CDSs (%)	rRNA genes (operons)	tRNA genes	scaffolds
FP830^T	6449812	5596062	61.0	5801	86.76	65	70	1
FP2034	6531494	5704413	60.0	5665	87.34	16	66	1
FP2262	6604522	5751993	60.9	5734	87.09	15	64	1

Table S3. The gene cluster type, location, compound, and size of secondary metabolites in FP830^T.

Cluster no.	Type	From	To	Size (nt)	Most similar known cluster	Similarity
1	NRPS-like	1159186	1194101	34915	Fragin	37%
2	Arylpolyene	1476310	1519921	43611	APE Vf	35%
3	RiPP-like	2397318	2406273	8955	Unknown	-
4	NRPS	2952023	3003775	51752	Pyoverdin	10%
5	Betalactone	3241336	3264645	23309	Fengycin	13%
6	Ranthipeptide	3579272	3600702	21430	Pyoverdin	8%
7	RiPP-like	2397318	2406273	8955	Unknown	-
8	NRPS	4216098	4266550	50452	Coelibactin	36%
9	NRPS	4659246	4734440	75194	Crochelin A	7%
10	Butyrolactone	4855354	4868764	13410	Unknown	-
11	NRPS	5323057	5402424	79367	Pyoverdin	20%
12	Lanthipeptide	5487209	5508696	21487	Unknown	-
13	Redox-cofactor	5826206	5848371	22165	Lankacidin C	13%

Table S4. Genes and gene clusters detected in FP830^T. genome predicted to be involved in plant growth-promoting activity and T3SS.

Locus tag	Gena name	Protein id	Protein coded by the gene or Predicted function
**Genes detected in FP830^T genome involved in the production of indole acetic acid (IAA)**			
PSH84_RS16080	*iaaH*	WP_305481365.1	Indoleacetamide hydrolase
PSH84_RS25960	*iaaM*	WP_305481983.1	Tryptophan 2-monooxygenase
**Genes detected in FP830^T genome involved in the production volatile compound (voc)**			
PSH84_RS23965	*ilvB*	WP_122564963.1	Acetolactate synthase isozyme 1 large subunit
PSH84_RS23960	*ilvN*	WP_003176102.1	Acetolactate synthase isozyme 1 small subunit
PSH84_RS17455	*ydjL*	WP_240998454.1	Uncharacterized zinc-type alcohol dehydrogenase-like protein
PSH84_RS20135	*budC*	WP_305481665.1	L-2,3-butanediol dehydrogenase
PSH84_RS17440	*acoA*	WP_122566440.1	2,6-dichlorophenolindophenol oxidoreductase subunit alpha
PSH84_RS17445	*acoB*	WP_122566441.1	2,6-dichlorophenolindophenol oxidoreductase subunit beta
PSH84_RS21990	*acoC*	WP_305467509.1	Cytoplasmic aconitate hydratase
PSH84_RS16280	*acoR*	WP_305470575.1	Acetoin dehydrogenase operon transcriptional activato
PSH84_RS20180	*bdh*	WP_305467178.1	1-butanol dehydrogenase
**Genes detected in FP830^T genome involved in the production of pyrroloquinoline quinone (PQQ)**			
PSH84_RS25980	*pqqB*	WP_122566669.1	Coenzyme PQQ synthesis protein B
PSH84_RS25985	*pqqC*	WP_305468686.1	Pyrroloquinoline-quinone synthase
PSH84_RS25990	*pqqD*	WP_122566667.1	PqqA binding protein
PSH84_RS17605	*pqqE*	WP_122566470.1	PqqA peptide cyclase
PSH84_RS26885	*pqqF*	WP_122567410.1	Coenzyme PQQ synthesis protein F
**Genes detected in FP830^T genome involved in the type II secretion system (T2SS)**			
PSH84_RS06840	*gspC*	WP_305482432.1	Protein N
PSH84_RS06845	*gspD*	WP_305482433.1	Secretin GspD
PSH84_RS06850	*gspE*	WP_305482434.1	ATPase GspE
PSH84_RS06855	*gspF*	WP_305482435.1	Inner membrane protein
PSH84_RS06865	*gspH*	WP_305482436.1	Minor pseudopilin GspH
PSH84_RS06870	*gspI*	WP_305482437.1	Minor pseudopilin GspI
PSH84_RS06875	*gspJ*	WP_305482438.1	Type II secretion system protein GspJ
PSH84_RS06880	*gspK*	WP_305482439.1	Type II secretion system protein GspK
PSH84_RS06885	*gspL*	WP_305482440.1	Type II secretion system protein GspL
PSH84_RS06890	*gspM*	WP_305482441.1	Type II secretion system protein GspM
**Genes detected in FP830^T genome involved in the type III secretion system (T3SS)**			
PSH84_RS27025	*hrpS*	WP_122567434.1	Transcriptional regulator
PSH84_RS27030	*hrpA*	WP_109752469.1	Structural component of pilus, type III secreted protein
PSH84_RS27040	*hrpB*	WP_122567435.1	Required for elicitation of HR and translocation and secretion of AvrPto1
PSH84_RS27045	*hrcJ*	WP_122567436.1	Putative connectors of the secretion apparatus across the periplasm
PSH84_RS27050	*hrpE*	WP_305468772.1	Required for elicitation of HR
PSH84_RS27065	*hrcC*	WP_122567440.1	Outer-membrane associated protein
PSH84_RS27070	*hrpT*	WP_122567441.1	Accessory protein, outer-membrane associated protein
PSH84_RS27075	*hrpV*	WP_122567442.1	Negative regular of hrp expression
PSH84_RS27090	*hrcU*	WP_305468775.1	Inner membrane associated protein
PSH84_RS27095	*hrcT*	WP_109752489.1	Inner membrane associated protein
PSH84_RS27100	*hrcS*	WP_003206603.1	Inner membrane associated protein
PSH84_RS27105	*hrcR*	WP_116833691.1	Inner membrane associated protein
PSH84_RS27110	*hrcQb*	WP_122567445.1	Inner membrane associated protein
PSH84_RS27115	*hrcQa*	WP_122567445.1	Inner membrane associated protein
PSH84_RS27125	*hrcN*	WP_305471289.1	Inner membrane associated protein,
PSH84_RS27130	*hrpQ*	WP_305468776.1	Similar to FliG, a cytoplasmic protein regulating flagellar biogenesis
PSH84_RS27135	*hrcV*	WP_122567449.1	Inner membrane associated protein
PSH84_RS27140	*hrpJ*	WP_122567450.1	Type III secreted protein for translocation of effectors
PSH84_RS27145	*hrpL*	WP_122567451.1	RNA polymerase sigma factor
PSH84_RS23340	*hrpK*	WP_305481870.1	Pathogenicity locus protein
**Genes detected in FP830^T genome involved in the type VI secretion system (T6SS)**			
PSH84_RS28045	*tssM*	WP_122567581.1	Membrane subunit
PSH84_RS28080	*tssC*	WP_122567584.1	Contractile sheath large subunit
PSH84_RS28085	*tssD*	WP_122567585.1	Tube protein
PSH84_RS28105	*tssF*	WP_122567588.1	Baseplate subunit
PSH84_RS28220	*tssF*	WP_122567608.1	Baseplate subunit
PSH84_RS28225	*tssE*	WP_122567609.1	Baseplate subunit
PSH84_RS28235	*tssB*	WP_122567611.1	Contractile sheath small subunit
PSH84_RS28240	*tssA*	WP_122567612.1	Protein TssA
PSH84_RS28075	*tssB*	WP_305482065.1	Contractile sheath small subunit
PSH84_RS28110	*tssG*	WP_305482067.1	Baseplate subunit
PSH84_RS28120	*tssI*	WP_305482068.1	Vgr family protein
PSH84_RS28140	*evpJ*	WP_305482071.1	RHS repeat-associated core domain-containing protein
PSH84_RS28180	*tssL*	WP_305482074.1	Type IVB secretion system protein IcmH/DotU
